# The Complete Mastoid Operation

**Published:** 1900-01-27

**Authors:** 


### THE COMPLETE MASTOID OPERATION.

At the meeting of the Royal Medical Chirurgical Society held on Tuesday, Mr. Ballance described the operation which he performs for the complete and permanent cure of chronic purulent otorrhœa. The operation is devised on ordinary surgical principles, and aims at opening up the whole of the pus producing cavity, doing away with pockets and recesses, the production of a clean surface, and the grafting of this surface so as to cover it all, even down to the bottom of the tympanum, with a smooth layer of epidermis. It will be understood that the cavity of the tympanum extends upwards far above the level that can be seen on looking down the meatus, and into it there opens the way into the mastoid antrum and the mastoid cells. What Mr. Ballance does is to entirely drill or gouge away the cancellous tissue which intervenes between the attic and the external surface of the bone, or, in other words, to enlarge the meatus upwards and backwards to such an extent as to throw all these cavities into one, and to lay the whole of the tympanum open to inspection, and then, after clearing out all pus-producing granulations, to cover the exposed surface of the bone with a graft, in a single piece if possible, so that there shall be left a clean cavity skinned over to the bottom. Very minute details were given as to the various steps of the two operations into which the process is divided —the one for the removal of the disease, and the other for the healing of the wound—and the various burrs and drills and gouges used were shown. After this Sir William Dalby made some remarks upon the selection of cases for operation, and Mr. A. E. Cumberbatch read a paper on the same subject. In the following groups of cases Mr. Cumberbatch thought that radical measures were either inexpedient, or should not be undertaken too hastily: (1) Cases in which, although the whole or the greater part of the membrana tympani has been destroyed, and the mucous lining of the tympanic cavity is greatly hypertrophied, in fact, is polypoid, there yet is no obvious bone disease. (2) Cases in which there is frequent recurrence of discharge on slight provocation, although the discharge is on each occasion readily arrested, perforation sometimes being permanent, and sometimes closing as soon as the discharge ceases. But among cases in this group, whenever the onset of the discharge is invariably preceded by malaise, slight headache, and rise of temperature, and occasional mastoid tenderness or discomfort, the operation should be performed. (3) Cases in which there is discharge from both ears, and in which the hearing is excellent, and there are no symptoms calling for operative interference beyond the discharge.

The discussion on these papers was very properly adjourned to a future meeting. The question of the treatment of otorrhœa is a most important one, for, as Sir William Dalby remarked, if the dictum is to be accepted that in all cases in which discharge continues and resists other treatment operation is required, the cases waiting for treatment, even in London alone, are to be numbered by thousands.

